# Time dependence of changes of two cartilage layers in anterior cruciate ligament insertion after resection on chondrocyte apoptosis and decrease in glycosaminoglycan

**DOI:** 10.1186/1758-2555-1-27

**Published:** 2009-12-10

**Authors:** Masataka Sakane, Hirotaka Mutsuzaki, Shinya Hattori, Hiromi Nakajima, Naoyuki Ochiai

**Affiliations:** 1 Department of Orthopaedic Surgery, Institute of Clinical Medicine, Graduate School of Comprehensive Human Science, University of Tsukuba, 1-1-1 Tennoudai, Tsukuba, Ibaraki 305-8575, Japan; 2 Department of Orthopaedic Surgery, Ibaraki Prefectural University of Health Sciences, 4669-2 Ami Ami-machi, Inashikigun, Ibaraki 300-0394, Japan; 3 Biomaterial Center, National Institute for Materials Science, 1-1 Namiki, Tsukuba, Ibaraki 305-0044, Japan; 4 Department of Agriculture, Ibaraki University, 3-21-1 Chuuou, Ami, Ibaraki 300-0393, Japan

## Abstract

**Background:**

The purpose of this study is to clarify the differences in time-dependent histological changes (chondrocyte apoptosis and glycosaminoglycan (GAG) layer thickness decrease) between uncalcified fibrocartilage (UF) and calcified fibrocartilage (CF) layers at the anterior cruciate ligament (ACL) insertion after ACL resection of rabbits.

**Methods:**

Forty male Japanese white rabbits underwent ACL substance resection in the right knee (resection group) and same operation without resection in the left knee (sham group). Animals were sacrificed 1, 2, 4 and 6 weeks after surgery.

**Results:**

In the UF layer, the apoptosis rate in the resection group was significantly higher than that in the sham group at 1 and 2 weeks. The GAG layer thicknesses of the UF layer in the resection group at 1, 2, 4 and 6 weeks were lower than those in the sham group. In the CF layer, the apoptosis rate in the resection group was significantly higher than that in the sham group at 2 and 4 weeks. The GAG layer thickness of the CF layer in the resection group was lower than that in the sham group only at 6 weeks.

**Conclusion:**

The increase in chondrocyte apoptosis rate preceded the decrease in GAG layer thickness in both layers. In the UF layer, the increase in chondrocyte apoptosis rate and the decrease in GAG layer thickness preceded those in the CF layer. Using a surviving ligament and minimizing a debridement of ACL remnant during ACL reconstruction may be important to maintain cartilage layers of ACL insertion. An injured ACL should be repaired before degenerative changes of the insertion occur.

## Introduction

An anterior cruciate ligament (ACL) insertion consists of four distinguishable tissue layers of transition, namely, ligaments, uncalcified fibrocartilage (UF), calcified fibrocartilage (CF), and bone [1-3]. Gradual hardness changes of different tissues reduce stress concentration at the insertion site [1,3]. Including glycosaminoglycan (GAG) in the cartilage zone provides water absorbability and flexibility to the ligaments [4]. The GAG and fibrocartilage layers are presumed to resist tensile, compressive and shear stresses at the insertion site [3]. An understanding of the structural property and extracellar matrix of the ACL-to-bone insertion site is necessary for the early management of ACL injury and regeneration of a soft-hard tissue interface after ACL reconstruction.

In a human ACL tibial insertion from 19 to 206 days after rupture, approximately 42% chondrocyte apoptosis rate and time-dependent degenerative histological changes of the cartilage layers have been observed in our previous report [5]. Moreover, we have reported that an increase in apoptosis rate preceded a decrease in GAG layer thickness at the CF layer in an ACL insertion, as determined using an ACL resection model in a small number of rabbits [6]. After ACL rupture, chondrocyte apoptosis can lead to degenerative changes of cartilage layers in the insertion. However, it is unclear that the differences in time-dependent histological changes between the UF and CF layers at the ACL insertion after rupture. It is hypothesized that the responses of the UF and CF layers in terms of histological changes are not the same, because of the differences in their mechanical properties and/or environment from synovial fluid. This is the first study to clarify the different responses of the UF and CF layers in an ACL insertion after ACL resection. The purpose of this study is to clarify the differences in time-dependent histological changes between the UF and CF layers at the ACL insertion after ACL resection of rabbits.

## Materials and methods

### Surgical procedure

Forty skeletally immature male Japanese white rabbits (weight range, 2.5-3.0 kg) were used for this study. The rabbits were maintained in accordance with the guidelines for the care and use of laboratory animals established by the Ethical Committee of the Department of Agriculture, Ibaraki University of Health (NIH Pub. No. 85-23 Rev. 1985). After receiving an intravenous injection of barbiturate (40 mg/kg body weight), an anterior lateral skin incision was made on the right knee. The ACL was then completely transected and gross anterior subluxation of the tibia was demonstrated by manual examination (resection group) and same operation without native ACL substance resection in the left knee (sham group) [6]. The incision area was sewed with 2-0 nonabsorbable sutures. After the operation, each animal was allowed free movement in a cage without receiving any antibiotics. Eight animals were euthanized with deep anaesthesia at five time periods (1, 2, 4 and 6 weeks) after surgery. We used normal ACL insertions as reference value (n = 8).

### Histomorphological analysis

ACL-tibia complexes were obtained from each animal's hind limb, which were trimmed to determine the sagittal plane of the central region of the insertion site. In the normal ACL insertions, right knee were used for this study. No substantial ACL remained. Specimens were fixed in 4% paraformaldehyde (pH 7.4) for one week. After fixation, all specimens were decalcified using 10% EDTA (pH 7.4) and embedded in paraffin. For each specimen, 5 μm-thick serial sections of the sagittal plane of the insertion site were stained with haematoxylin-eosin (HE). Safranin-O staining was conducted to observe glycosaminoglycan (GAG) and terminal deoxynucleotidyl transferase-mediated deoxyuridine triphosphate-biotin nick-end labeling (TUNEL) to detect apoptotic cells in the ACL insertion.

TUNEL was carried out in accordance with the instruction included in the Apoptag^® ^Plus Peroxidase *In Situ *Apoptosis Detection kit (Chemicon International Inc., USA and Canada) except for counterstaining. Using the Apoptag method, the sections were incubated in an equilibrium buffer for 10 min at room temperature. After that, terminal deoxynucleotidyl transferase (TdT) and digoxigenin-labeled nucleotides were incubated for 60 min at 37°C in a humid chamber. Then, a peroxidase-conjugated digoxigenin antibody was incubated with the sections for 30 min at room temperature in the humid chamber. After the immunoreaction product was developed in diaminobenzidine, the sections were counterstained with Mayer's haematoxylin for 30 s. TUNEL-positive nuclei stained dark brown and TUNEL-negative nuclei stained blue.

**Figure 1:**
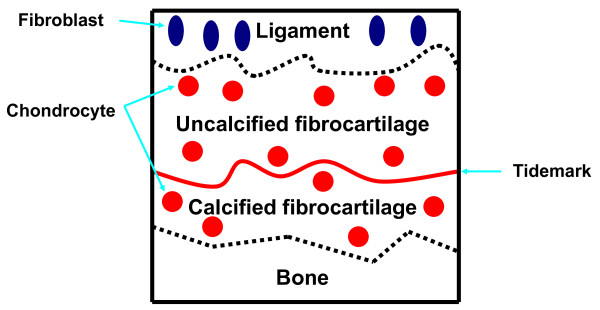
**Schema of tibial insertion**. The UF layer is defined as the area between the ligament and the tidemark. The CF layer is defined as the area between the tidemark and the lamellar bone.

Histomorphometrical analysis method was based on that used in our previous study [5,6]. The sections were examined under a light microscope (BX-51, Olympus Optical Co., Ltd., Tokyo, Japan) equipped with a CCD camera system (DP50, Olympus). The UF layer was distinguished between ligaments in terms of cell shape and the aspect of the extracellular matrix by HE staining. Moreover, the UF layer was also distinguished from the CF layer with reference to the tidemark by HE staining. We measured the area of the Safranin-O-stained GAG layer in the UF layer between the ligament and tidemarks by HE staining and Safranin-O staining. Because the CF layer between the tidemark and the bone was a distinct structure, we measured the GAG-stained area in the CF layer between the tidemark and the lamellar bone by HE staining and Safranin-O staining (Fig. 1). Using a Mac Scope program (Mitani Co., Japan) on a Macintosh computer, the percentage of TUNEL-positive chondrocytes and the GAG-stained area of UF and CF layers were determined. The GAG-stained area and the numbers of chondrocytes and TUNEL-positive chondrocytes in the UF and CF layers were measured. The obtained GAG-stained area was divided by the width of each ACL tibial insertion, and the value obtained was defined as the average thickness of the GAG-stained area in the UF and CF areas. The average percentage of TUNEL-positive chondrocytes was calculated from the number of TUNEL-positive chondrocytes divided by that of all chondrocytes in the UF and CF areas.

### Statistical analysis

Concerning the average percentage of TUNEL-positive chondrocytes and the average thickness of GAG-stained area in the UF and CF areas, statistical analysis of differences between the resection and sham groups at the same period was performed using a paired t-test. To examine the time-dependent histological changes, a one-way analysis of variance (ANOVA) was performed on data of the resection and sham groups. The significance was set at 5%. On the factors found to be significantly different by ANOVA, the Tukey-Kramer test was performed.

## Results

**Figure 2:**
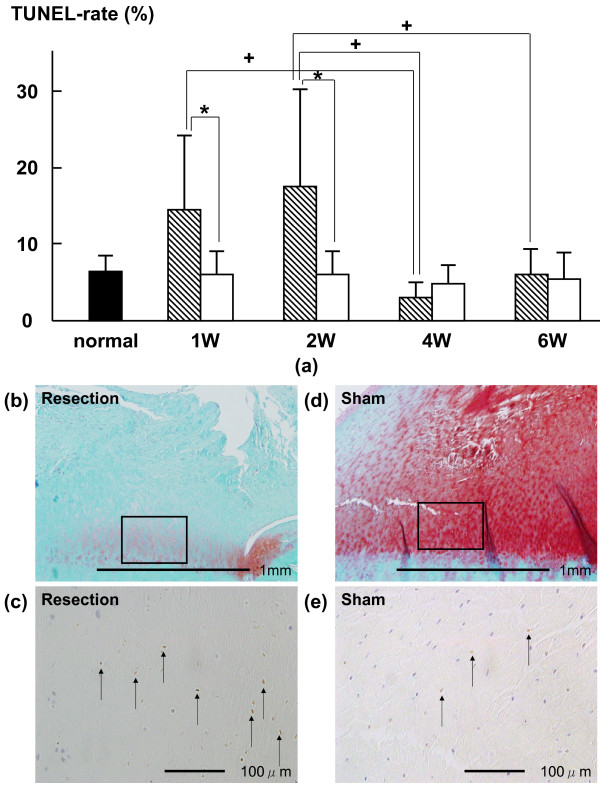
**(a) Time-dependent changes in average percentage of TUNEL-positive chondrocytes in the UF layer after surgery**. The average percentages of TUNEL-positive chondrocytes in the resection group at 1 and 2 weeks were significantly higher than those in the sham group. The average percentage of TUNEL-positive chondrocytes at 1 week was significantly higher than that at 4 weeks. The average percentage of TUNEL-positive chondrocytes at 2 weeks was significantly higher than those at 4 weeks and 6 weeks. Shaded bars: Resection group, Unshaded bars: Sham group Safranin-O staining of histological sections of at 2 weeks in UF layer; (b): resection group, (d): sham group. (c) and (e) are magnified views of the boxed part in (b) and (d) that are TUNEL of histological sections at 2 weeks in UF layer. The TUNEL-positive chondrocytes are brown (arrow).

**Figure 3:**
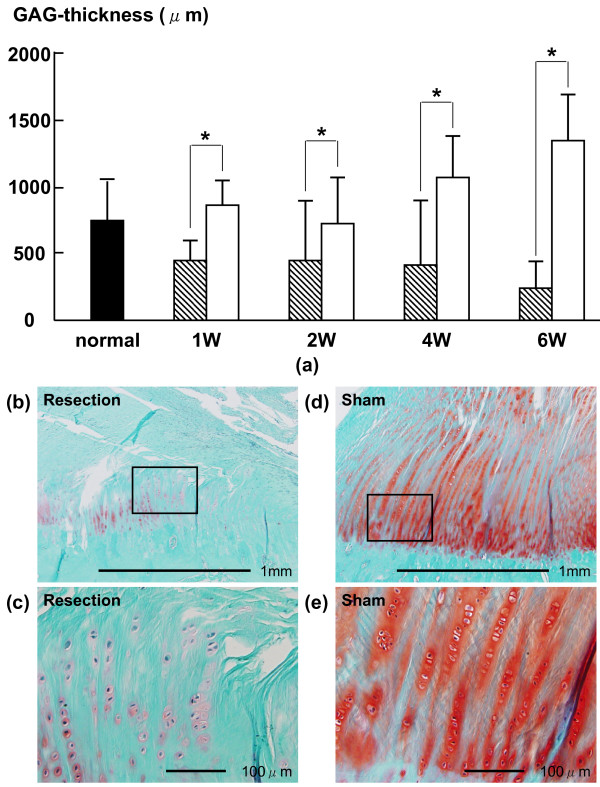
**(a) Time-dependent changes in average thickness of GAG-stained area in UF layer**. The average thicknesses of the GAG-stained area in the resection group at 1, 2, 4 and 6 weeks were lower than those in the sham group. Shaded bars: Resection group, Unshaded bars: Sham group Safranin-O staining of histological sections of at 4 weeks in UF layer; (b): resection group, (d): sham group. (c) and (e) are magnified views of the boxed part in (b) and (d). The GAG staining density in the resection group was lower than that in the sham group.

**Figure 4:**
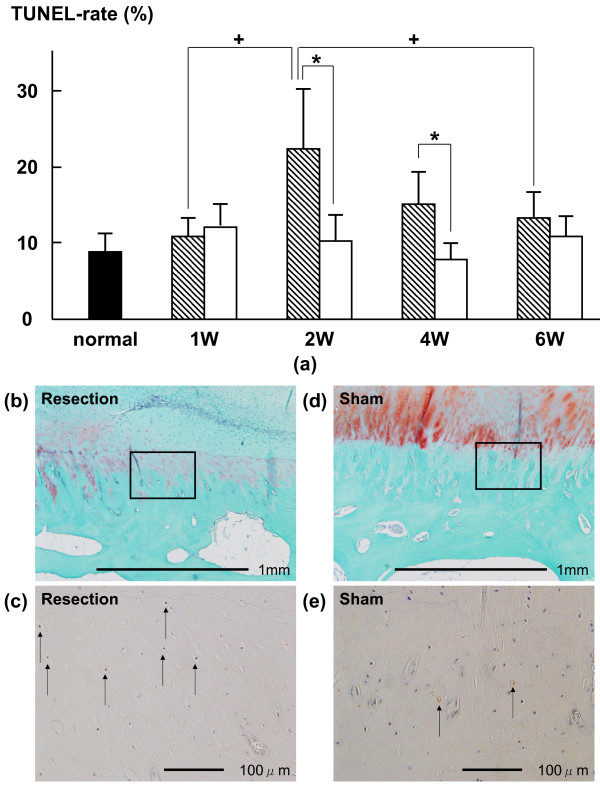
**(a) Time-dependent changes in average percentage of TUNEL-positive chondrocytes in CF layer after surgery**. The average percentages of TUNEL-positive chondrocytes in the resection group at 2 and 4 weeks were significantly higher than those in the sham group. The average percentage of TUNEL-positive chondrocytes at 2 week was significantly higher than those at 1 and 6 weeks. Shaded bars: Resection group, Unshaded bars: Sham group Safranin-O staining of histological sections of at 4 weeks in CF layer; (b): resection group, (d): sham group. (c) and (e) are magnified views of the boxed part in (b) and (d) that are TUNEL of histological sections at 4 weeks in CF layer. The TUNEL-positive chondrocytes are brown (arrow).

**Figure 5:**
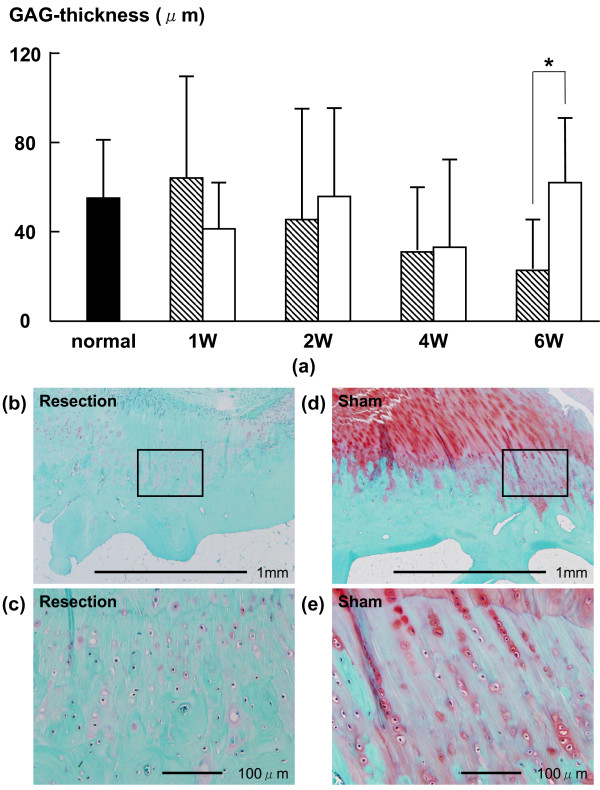
**Time-dependent changes in average thickness of GAG-stained area in CF layer**. The average thickness of the GAG-stained area in the resection group at 6 weeks was lower than that in the sham group. Shaded bars: Resection group, Unshaded bars: Sham group Safranin-O staining of histological sections of at 6 weeks in CF layer; (b): resection group, (d): sham group. (c) and (e) are magnified views of the boxed part in (b) and (d). The GAG staining density in the resection group was lower than that in the sham group.

In the UF layer, the average percentages of TUNEL-positive chondrocytes in the resection group at 1 and 2 weeks (14.1 ± 10.6% (p = 0.0061) and 17.6 ± 12.1% (p = 0.0166), respectively) were higher than those in the sham group (5.6 ± 3.7% and 5.5 ± 3.6%, respectively) (Fig. 2). The average thicknesses of the GAG-stained area of the UF layer in the resection group at 1, 2, 4 and 6 weeks (471.0 ± 185.3 (p = 0.0039), 444.7 ± 429.5 (p = 0.0393), 382.2 ± 503.1 (p = 0.0192) and 225.9 ± 258.7 (p = 0.0001) μm, respectively) were lower than those in the sham group (836.0 ± 269.1, 729.6 ± 423.0, 1069.1 ± 371.1 and 1341.1 ± 406.2 μm, respectively) (Fig. 3). In the CF layer, the average percentages of TUNEL-positive chondrocytes in the resection group at 2 and 4 weeks (22.6 ± 9.3% (p = 0.0082) and 15.0 ± 5.4% (p = 0.0015), respectively) were higher than those in the sham group (10.2 ± 4.8% and 7.5 ± 2.8%, respectively) (Fig. 4). The average thickness of the GAG-stained area in the resection group (23.0 ± 23.9 μm) at 6 weeks was lower than that in the sham group (62.7 ± 32.0 μm (p = 0.0152)) (Fig. 5).

In the UF layer of the resection group, the average percentage of TUNEL-positive chondrocytes at 1 week (14.1 ± 10.6%) was significantly higher than that at 4 weeks (2.6 ± 2.2%, P < 0.05). In the UF layer of the resection group, the average percentage of TUNEL-positive chondrocytes at 2 weeks (17.6 ± 12.1%) was significantly higher than those at 4 weeks and 6 weeks (2.6 ± 2.2% (P < 0.01) and 5.6 ± 3.3% (P < 0.05), respectively) (Fig. 2). The average thickness of the GAG-stained area in the UF layer of the resection group was not significantly different among all phases (Fig. 3).

In the CF layer of the resection group, the average percentage of TUNEL-positive chondrocytes at 2 weeks (22.6 ± 9.3%) was significantly higher than those at 1 week and 6 weeks (10.7 ± 2.8% (P < 0.01) and 13.2 ± 4.5% (P < 0.05), respectively) (Fig. 4). The average thickness of the GAG-stained area of the CF layer in the resection group was not significantly different among all phases (Fig. 5). All histomorphometrical data are summarized in Table 1.

**Table 1:** Histomorphometrical data

			normal ACL	1W	2W	4W	6W
TUNEL-rate (%)	UF	Resection	6.6 ± 3.1	14.1 ± 10.6 *+	17.6 ± 12.1 *+	2.6 ± 2.2 +	5.6 ± 3.3 +
				
		Sham		5.6 ± 3.7 *	5.5 ± 3.6 *	4.8 ± 2.8	5.3 ± 3.5
	
	CF	Resection	8.7 ± 2.6	10.7 ± 2.8 +	22.6 ± 9.3 *+	15.0 ± 5.4 *	13.2 ± 4.5 +
				
		Sham		12.3 ± 3.1	10.2 ± 4.8 *	7.5 ± 2.8 *	10.6 ± 4.3

GAG-thickness (μm)	UF	Resection	742.8 ± 337.6	471.0 ± 185.3 *	444.7 ± 429.5 *	382.2 ± 503.1 *	225.9 ± 258.7 *
				
		Sham		836.0 ± 269.1 *	729.6 ± 423.0 *	1069.1 ± 371.1 *	1341.1 ± 406.2 *
	
	CF	Resection	53.8 ± 28.2	64.8 ± 50.2	46.0 ± 52.8	32.2 ± 29.7	23.0 ± 23.9 *
				
		Sham		40.9 ± 21.8	56.6 ± 41.0	33.0 ± 40.3	62.7 ± 32.0 *

*-indicate a significant difference between groups in the same week (P < 0.05).

+ indicates a significant difference between weeks in the same group (P < 0.05).

## Discussion

In this study, we clarified that chondrocyte apoptosis can lead to a degenerative change (decrease in GAG layer thickness) in both cartilage layers, namely, the UF and CF layers in the ACL insertion after resection. The chain of events observed in this study was similar to that observed in our previous study of only the CF layer using a small number of rabbits [6]. Moreover, we newly clarified the chain of events: that is, the increase in chondrocyte apoptosis rate and decrease in GAG layer thickness in the UF layer preceded those in the CF layer. In the UF layer, the increase in chondrocyte apoptosis rate was observed at 1 and 2 weeks, and the decrease in GAG layer thickness was observed from 1 to 6 weeks. On the other hand, in the CF layer, the increase in chondrocyte apoptosis rate was observed at 2 and 4 weeks, and the decrease in GAG layer thickness was observed at 6 weeks. Time-dependent differences in the chain of events between the UF and CF layers might be due to differences of tissue hardness, differences in the transmission order of mechanical stress, changes in the mechanical transmission mode after resection, and differences in sensitivity to cytokines from synovial fluid.

The apoptotic cell rate range is 3-10% in an intact articular cartilage in adult rats and mice [7]. An enhanced chondrocyte apoptosis can cause imbalances in the levels of matrix metalloproteinases and their inhibitor derived from affected synovial membranes [8-10], and in proinflammatory responses [11]. In the acute and subacute phases after injury, inflammatory cytokines are considered to affect chondrocyte apoptosis and matrix degradation [11,12]. Additionally, mechanical factors such as knee instability and/or stress deprivation [13-15], also affect apoptosis in tissues. In the sham group, a peak in chondrocyte apoptosis rate was not observed, and the decrease in GAG layer thickness was not observed, either. We considered that histological changes do not occur in association with proinflammatory responses to the surgical in the sham group.

Clinically, the understanding of differences in time-dependent changes between the UF and CF layers is important for regeneration a soft and hard-tissue interface after ACL reconstruction and for rehabilitation after ACL repair. At the bone-tendon junction with an implanted a soft tissue graft in a bone tunnel, UF may regenerate earlier than CF. Using a surviving ligament and minimizing a debridement of ACL remnant during ACL reconstruction may be important to maintain cartilage layers of ACL insertion. An injured ACL should be repaired before degenerative changes of the insertion occur. At the ACL insertion after ACL repair, UF may react earlier than CF. Probably, the regeneration capability of the insertion may be different between the two cartilage layers. An in-depth understanding of an injured ACL insertion may help elucidate the etiology of the histological changes of the insertion, and may help in devising optimal treatment protocols for ACL injuries if apoptosis is controlled. In the future, immunohistochemical and mechanical analyses are necessary for defining key factors for differentiating between the UF and CF layers in not only ACL insertions but also other insertions to regenerate the soft-hard tissue interface.

## Competing interests

The authors declare that they have no competing interests.

## Authors' contributions

MS conceived of the study, and participated in its design and coordination. HM performed the statistical analysis, participated in the sequence alignment and drafted the manuscript. SH and HN carried out the histological analysis. NO participated in the sequence alignment. All authors read and approved the final manuscript.
